# Coming together to document mortality in conflict situations: proceedings of a symposium

**DOI:** 10.1186/1752-1505-3-2

**Published:** 2009-02-25

**Authors:** Ruwan Ratnayake, Olivier Degomme, Debarati Guha-Sapir

**Affiliations:** 1 WHO Collaborating Centre for Research on the Epidemiology of Disasters, Université catholique de Louvain School of Public Health, 30 94 Clos Chapelle aux Champs, 1200 Brussels, Belgium

## Abstract

The use of epidemiology in documenting the mortality experience in complex emergencies has become pervasive in humanitarian practice. Recent assessments in Iraq and Darfur have provoked much discussion on the assessment of mortality in scientific and policy spheres. In this context, the Centre for Research on the Epidemiology of Disasters and the Harvard Humanitarian Initiative held an inter-disciplinary symposium to examine the topic among epidemiologists, demographers, forensic scientists and legal and human rights investigators.

We aimed to strengthen the scientific understanding of mortality estimation by reviewing progress across fields and building inter-disciplinary bridges. We report on the presentations and discussions here.

## Introduction

The use of epidemiology in documenting the mortality experience of complex emergencies has become pervasive across humanitarian practice. Although used primarily as an operational tool, in recent years epidemiological practice has been placed front and centre in the larger debates over the deaths of civilians in Darfur and Iraq. Several other approaches have long been used to assess mortality in conflict settings, including forensic analyses of causes of death and investigations into the abuses of those who have perished.

This overlap is not exactly a coincidence. Mortality is the ultimate indicator of human health and has wide-ranging implications on the understanding of the scale of a crisis on a population, the use of violence against civilians and culpability. But how can disciplines such as epidemiology, demography, statistics, law, human rights documentation and forensic science best coordinate with each other to describe the mortality experience of civilian populations? Each field has distinct objectives. There is a need to develop an understanding of these objectives to ultimately provide a coherent understanding of various numerical estimates and accounts. This will improve scientific communication and prevent confusion among the end users of these estimates.

On November 6^th ^and 7^th^, 2008, the Centre for Research on the Epidemiology of Disasters (CRED) together with the Harvard Humanitarian Initiative (HHI) held a breakthrough symposium in Brussels, Belgium to open the dialogue between disciplines [1]. The main objectives were to strengthen the scientific basis of mortality documentation by drawing on recent progress in the disciplines of field epidemiology, demography, forensic science, statistical analysis and human rights investigation and to build bridges between disciplines for improved and reliable estimation.

Although meetings within each discipline have been held previously, we believe that this is the first to bring such a diverse array of actors together. An important precursor to this meeting was held in 1998 by the U.S. National Academy of Sciences to review the then nascent field of mortality estimation among displaced populations [2]. For the current meeting, CRED and HHI brought together forty discussants from thirty organizations representing academia, non-governmental organizations, international organizations, governments and United Nations agencies (see Appendix 1 for full list).

## Which methods are used?

The sessions explored various topics concerning methods and applications. The broad objectives of estimation and documentation were first laid out. Pierre Salignon from the Health and Nutrition Tracking Service, an inter-agency initiative for the generation of coherent data across humanitarian emergencies, discussed the role of mortality data in advocating for populations in crisis [3]. He described Médecins Sans Frontières' efforts to systematically document violence against civilian populations in Congo-Brazzaville in 2000. Helge Brunborg from Statistics Norway then explained the role of the demographic estimation of mortality for judicial purposes with reference to his work with the International Criminal Tribunal for the Former Yugoslavia (ICTY) [4]. Through an operational perspective gained from missions with the World Health Organization and the International Rescue Committee, Les Roberts emphasized the use of mortality data for the direction of humanitarian health programs in the Democratic Republic of Congo and other key crises [5]. This session provided a framework for a cross-disciplinary discussion of methods and challenges to investigations.

The methods used to collect primary data vary by the point in time during which they are applied. Overviews of 'in situ' methods conducted during a conflict and challenges posed by these methods were given. Presenters described retrospective sample surveys conducted primarily by nongovernmental organizations for operational purposes (Olivier Degomme, CRED), prospective surveillance systems maintained during a war in Guinea-Bissau (Jens Nielsen, Bandim Health Project) and the use of witness accounts to highlight human rights violations among Darfurian refugees in Chad (Jan Pfundheller, Atrocities Documentation Team) [6-8].

Forensic investigation, which can elucidate violent causes of death and age and sex characteristics of victims, can be applied at the point where human remains can be carefully recovered. The application of forensic approaches was described using the cases of mass gravesites in Bosnia and Herzegovina (in Praća and Rahunići), Sri Lanka and across Guatemala (Tal Simmons, University of Central Lancashire and Fredy Peccerelli, Guatemalan Forensic Anthropology Foundation) [9,10].

Researchers may take a more thorough look at the larger mortality experience using secondary data collected at an earlier point in time. Aggregation and tallying methods can be used to draw a cohesive picture of mortality estimations across a particular crisis. The amassed results of field surveys conducted at sub-national geographical areas (Olivier Degomme, CRED) and at the global level through the multi-country, retrospective Demographic and Health Surveys and World Health Surveys conducted by UN agencies (Ziad Obermeyer, Institute for Health Metrics and Evaluation) can be used to explore trends at a national level [11,12]. Challenges to the use of secondary data, precision and biases inherent to surveys in conflict areas were outlined. Another approach, multiple systems estimation, also uses multiple though disparate data sources (including surveys, qualitative testimonies and graveyard censes) to compare single-source mortality estimations. Its use to estimate deaths in Timor-Leste between 1975 and 1999 was described (Romesh Silva, Benetech) [13].

Investigators have been innovative in their use of both conventional and new technologies to make estimations in specific crises. The use of databases of validated media reports in the current Iraq conflict (Hamit Dardagan, Iraq Body Count), extensive reviews of state of the art mortality data sources for prosecution purposes at the International Criminal Court (ICC) (Guillermo Bedoya Jimenez, ICC) and satellite imagery to assess the extent of the burning of villages in Darfur (Phil Clarke, Bloodhound) were discussed [14-16]. These approaches represent important developments in addressing mortality estimation in areas where humanitarian access is poor, rigorous data collection is problematic and questions of the geographical distribution of mortality persist.

## What are the pitfalls and limitations of mortality documentation?

Whether using primary or secondary data, methodological issues in the collection and analysis of data collected during active conflict are inherent to the exercise. With reference to the demographic analysis of mortality in Iraq, the duration of the war is difficult to pinpoint, credible baseline mortality rates may be unavailable and estimations of the base population are greatly affected by the available data on demographic changes (Beth Osborne Daponte, Yale University) [17]. Similarly, questions remain as to the acceptable precision of well-used sampling designs in the estimation of violent deaths, as demonstrated by an analysis of the results of the 2004 Iraq Living Conditions Survey (Michael Spagat, University of London) [18].

The collection of data in the field can be an extremely difficult logistical challenge. Surveys of the affected crisis regions of Darfur, Sudan by the World Health Organization and the Democratic Republic of Congo by the International Rescue Committee (IRC) have faced security and logistical obstacles that intensify the methodological limitations and risk to personnel, hinder the implementation of best practices and ensure that difficult choices must be made throughout the data collection period (Alessandro Colombo, IRC) [19]. Similarly, personal risk may be extended outside of the field situation due to the nature of the inquiry. Threats to the lives of investigators have persisted during forensic investigations in Guatemala (Fredy Peccerelli, Guatemalan Forensic Anthropology Foundation) [20]. In both cases, investigators and field personnel require skills in negotiation and an acute sensitivity to the political environment.

The application of data to humanitarian interventions also faces obstacles. Statistical issues in the evaluation of interventions to reduce mortality are rendered difficult in the absence of a control group that is unaffected by conflict (Jens Nielsen, Bandim Health Project) [21]. Data collected in order to understand the political trajectory of violence, such as in the widely-used Centre for Civil War/PRIO battle deaths dataset, has less immediate use for humanitarian programming (Bethany Lacina, Stanford/PRIO) [22]. Though it may serve as a valuable evidence for humanitarian intervention, the utilization of mortality data to make timely policy decisions is dependent on the available data which may be anecdotal, unsound or unrepresentative (Mark Phelan, U.S. Department of State) [23].

## How can we move forward?

The symposium clearly opened more doors and derived more questions than could be adequately explored over two days. Participants carefully reflected on avenues for collaboration among disciplines, to move past speculative discussions and attempt to put new thoughts to practice.

Collaboration in this case can be a difficult proposition. Debarati Guha-Sapir, of CRED, stated that it is relatively easy and scientifically safe to stay within one's bounds by avoiding inter-disciplinary collaboration. Les Roberts remarked that collaboration may face significant impediments due to the core objectives of different fields. For instance, the public health approach utilizes epidemiological tools to derive aggregated, confidential mortality data at the population level for the objective of directing humanitarian programmes. The judicial needs however, may require that mortality data is substantiated by the identification of victims. There also exist tradeoffs in committing resources for the extensive documentation of mortality for legal purposes as done for the ICTY versus obtaining a range of precision for the purpose of humanitarian practice.

'Serendipity', or the act of accidentally discovering something fortunate, is the other side to the coin. Catrien Bijleveld, a criminologist with VU University Amsterdam, used this word to describe discussions throughout the two days [24]. Participants remarked that the level of detail in the cost-effective satellite imagery presented by Bloodhound could greatly inform the interpretation of their own epidemiological findings. Most discussants did not have significant experience with forensic investigation. The application of this science to adequately determine the age, sex and cause-of-death among samples of victims can fill important voids in the interpretation of data for other disciplines where this information is difficult to obtain. Several other examples, relating to methodological issues between epidemiology and statistics were recognized as representative of larger issues within the core disciplines.

War itself will always be a divisive and value-laden issue. The field of public health has been reticent to acknowledge its specific ability to address conflict [25]. More recently, conflict epidemiology has emerged as its own discipline, and it follows that the debates of civilian deaths in Darfur and Iraq have truly reached the broadest levels of political, scientific and media discourse. Given this intense environment for scientific progress, responsibility for good quality data and the potential impact on human well-being, it is unsurprising that the symposium fostered healthy debate and genuine tensions over the core scientific approaches for mortality estimation. Mutual respect for scientific disciplines is imperative though respectful debates are valuable. Upon reflection, one participant summarized the reality which underlies this tension and hence the basis for such a symposium: 'there is no incompatibility here; [debate] is the nature of science'.

## Competing interests

The authors declare that they have no competing interests.

## Authors' contributions

RR drafted the report. All of the authors organized the symposium, contributed to revising the manuscript and gave final approval of the manuscript.

## Appendix

### Appendix 1: organizations represented

Amnesty International

Atrocities Documentation Team for Darfur

Bandim Health Project, Statens Serum Institut

Benetech Human Rights Data Analysis Group

Bloodhound

Brigham and Women's Hospital, Harvard University

Bureau of Population, Refugees, and Migration, U.S. Department of State

Centre for Research on the Epidemiology of Disasters

Deutsches Institut für Wirtschaftsforschung (DIW Berlin)

Epicentre

Guatemalan Forensic Anthropology Foundation

Harvard Humanitarian Initiative

Health and Nutrition Tracking Service

Households in Conflict Network

Independent science writer

Institute for Health Metrics and Evaluation, University of Washington

Institute for Social and Policy Studies, Yale University

International Criminal Court

International Peace Research Institute Oslo (PRIO)

International Rescue Committee

Iraq Body Count

Médecins Sans Frontières Belgium, France and UK

Netherlands Interdisciplinary Demographic Institute

Program on Forced Migration and Health, Columbia University

Royal Holloway College, University of London

School of Forensic and Investigative Sciences, University of Central Lancashire

Small Arms Survey

Special Court for Sierra Leone

Stanford University

Statistics Norway

Trinity College Dublin

Université catholique de Louvain

University of Antwerp

VU University Amsterdam

World Health Organization
